# Mechanical and Electrical Properties of Epoxy Composites Modified by Functionalized Multiwalled Carbon Nanotubes

**DOI:** 10.3390/ma14123325

**Published:** 2021-06-16

**Authors:** Paweł Smoleń, Tomasz Czujko, Zenon Komorek, Dominik Grochala, Anna Rutkowska, Małgorzata Osiewicz-Powęzka

**Affiliations:** 1 Institute of Materials Science and Engineering, Military University of Technology, Gen. Sylwestra Kaliskiego Str. 2, 00-908 Warsaw, Poland; zenon.komorek@wat.edu.pl; 2 Smart Nanotechnologies S.A., Karola Olszewskiego Str. 25, 32-566 Alwernia, Poland; anna.rutkowska@smartnanotech.com.pl; 3 Department of Electronics, AGH University of Science and Technology, Adama Mickiewicza Str. 30, 30-059 Cracow, Poland; grochala@agh.edu.pl; 4 New Era Materials Sp. z o.o., Komandosów Str. 1/7, 32-085 Modlniczka, Poland; malgorzata.osiewicz@neweramaterials.com

**Keywords:** polymer composites, multiwall carbon nanotubes, electrical properties, mechanical properties, functionalization, epoxy

## Abstract

This paper investigates the effect of multiwalled carbon nanotubes on the mechanical and electrical properties of epoxy resins and epoxy composites. The research concerns multiwalled carbon nanotubes obtained by catalytic chemical vapor deposition, subjected to purification processes and covalent functionalization by depositing functional groups on their surfaces. The study included the analysis of the change in DC resistivity, tensile strength, strain, and Young’s modulus with the addition of carbon nanotubes in the range of 0 to 2.5 wt.%. The effect of agents intended to increase the affinity of the nanomaterial to the polymer on the aforementioned properties was also investigated. The addition of functionalized multiwalled carbon nanotubes allowed us to obtain electrically conductive materials. For all materials, the percolation threshold was obtained with 1% addition of multiwalled carbon nanotubes, and filling the polymer with a higher content of carbon nanotubes increased its conductivity. The use of carbon nanotubes as polymer reinforcement allows higher values of tensile strength and a higher strain percentage to be achieved. In contrast, Young’s modulus values did not increase significantly, and higher nanofiller percentages resulted in a drastic decrease in the values of the abovementioned properties.

## 1. Introduction

Polymer nanocomposites are materials that consist of two or more phases (continuous and discontinuous) with a distinct interaction surface, of which at least one component has at least one dimension that is nanometric in scale. In contrast to conventional polymer composites based on micron-scale modifiers, the introduction of nanomaterials into polymers allows for small filler distances, so that composite properties can be modified to a large extent even at very low additive contents [1,2]. The nanofillers used can be divided according to their chemical properties (inorganic and organic), physical structure (crystalline, amorphous, and gas inclusions), and particle shape (three-, two- and one-dimensional). Depending on the type of polymer and nanofiller, polymer nanocomposites have applications as structural, functional, and coating materials [3]. The key aspect related to the effective use of nanomaterials in polymeric materials is their homogeneous dispersion in the matrix while maintaining strong interaction and adhesion between the polymer chain and nanofiller. The size of the contact surface of the discontinuous phase (filler), as well as the nature of the interactions between the continuous and discontinuous phases, plays a vital role [4,5].

Carbon nanotubes are one-dimensional materials characterized by a high length-to-diameter ratio that can be as high as 1000. They are made of rolled single sheets of graphite, referred to as graphene layers, that are a single layer of graphite-structured carbon atoms forming six-membered rings of carbon atoms with sp^2^ hybridization [6,7,8]. Their unique structure also gives unique properties, e.g., good mechanical and electrical properties, and taking into account other properties, such as low density, high expansion coefficient, or large specific surface area, they might be considered interesting reinforcing materials for composite materials [4,7].

An improvement in the mechanical and electrical properties is obtained when the length-to-diameter ratio of the filler increases, and when its transverse dimension decreases. In this case, the specific surface area of the filler increases, and thus, the interaction between the matrix and its particles increases [3]. Adhesion between the nanofiller and the polymer can be improved by functionalization of carbon nanotubes [9,10]. In the case of carbon nanotubes, the most common methods for the surface modification are physicochemical methods, which include covalent and noncovalent interactions [11].

Grafting of carboxyl groups, fluorinated nanotubes and nanotubes functionalized with isocyanates, amino groups, or biomolecules is often used to improve interactions and mechanical properties [6]. A number of review papers presenting the current knowledge on the mechanical properties of polymers with carbon nanotubes have been written [5,12]. The effect of the addition of carbon nanotubes on the mechanical properties has been discussed in detail in the work of Coleman et al. [13], who analyzed the influence of the composite processing method on the strength parameters of the composites obtained, while the work of Domun et al. [11] focused on the issue of improving the fracture toughness and strength of epoxy resin using nanomaterials, particularly carbon nanotubes.

Gojny [14] carried out an extensive comparative study that involved the changes in mechanical properties through the use of single-, double-, and multiwalled carbon nanotubes modified with amino groups, and examined the effect of adding carbon nanotubes to an epoxy resin. The use of double-walled carbon nanotubes with amino groups resulted in an improvement in tensile strength by 10% and Young’s modulus by 15%. On the other hand, Spitalsky’s research [15] proved that the method of dispersing 0.5% by weight of multiwalled carbon nanotubes in a solvent improved the tensile strength by 62% with an improvement in Young’s modulus by 54%. The effect of increasing the strength of epoxy composites has also been attempted using oxidized carbon nanotubes. In the work of Gou et al., a 40% increase in tensile strength and a 10% decrease in Young’s modulus values were reported using 4 wt.% carbon nanotubes introduced into the matrix using the stir method [16]. An opposite effect was obtained by Breton [17]—at 6% addition, a 32% increase in Young’s modulus and a 25% decrease in tensile strength were obtained.

**Figure 1:**
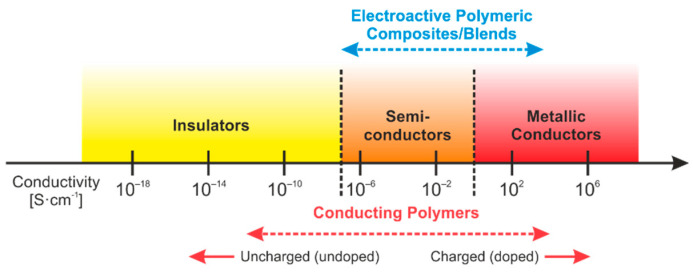
Conductivity range of conducting polymers and conductive materials based on [19].

The potential of carbon nanotubes is also often used in studies of the electrical properties of polymer nanocomposites. Carbon nanotubes, due to their specific structure and excellent electrical conductivity, make it possible to obtain a significant improvement in the electrical conductivity of polymers at a very low-weight fraction. A careful analysis of the literature indicates a large variation in the values of the electrical properties, as the electrical conductivity and the percolation threshold are highly dependent on many factors, including the type of polymer, the method of nanocomposite synthesis, the length-to-diameter ratio of the carbon nanotube, the degree of dispersion, or the functionalization of the additive [5,18]. Figure 1 presents conductivity ranges of conducting polymers and also conductive materials.

Establishing epoxy composites as the focal point of their study, Sandler et al. presented an improvement in electrical conductivity with 0.0025% unmodified carbon nanotubes with a length-to-diameter ratio of 340 added by hot shearing [20]. On the other hand, in the work of Kovacs [21], the percolation threshold was reached at 0.011% and 0.08% of unmodified carbon nanotubes using slow and fast shear mixing, respectively, proving that a slower mixing speed significantly increased the conductivity at the same length-to-diameter ratio of carbon nanotubes.

On the other hand, carbon nanotube dispersions prepared by sonication and mixing in epoxy resin resulted in obtaining carbon nanotubes with a low length-to-diameter ratio, resulting in conductivity thresholds at 5% carbon nanotubes [22]. On the other hand, chemical functionalization of the carbon nanotube surface with amino groups allows the percolation characteristics to be maintained; however, a lower conductivity was observed than that of the chemically untreated carbon nanotubes, obtaining a percolation threshold of 0.25% [23].

In view of the small amount of available literature regarding the effect of multiwalled carbon nanotubes modified with amine groups on the electrical and mechanical properties of thermoset polymers, as well as the many discrepancies in the currently reported results, we present research on the influence of amino-modified carbon nanotubes on the mentioned properties. Additionally, this paper presents the effect of nanoadditives obtained by the developed synthesis method on two pure epoxy resins and resins with additives used in industry, ensuring improved processing.

In this work, we present the structure, mechanical, and electrical properties of epoxy composites modified with multiwalled carbon nanotubes. Multiwalled carbon nanotubes were obtained by catalytic chemical vapor deposition, subjected to purification processes and covalent functionalization by depositing functional groups on their surfaces. The study included the analysis of the change in DC resistivity, tensile strength, strain, and Young’s modulus with the addition of carbon nanotubes in the range of 0 to 2.5 wt.%. The effect of agents causing an increase in the affinity of the nanomaterial to the polymer on the abovementioned properties was also investigated.

## 2. Materials and Methods

### 2.1. Functionalization of Carbon Nanotubes

Nanocyl NC7000 multiwalled carbon nanotubes were used as the filler for the polymer matrix. Carbon nanotubes were prepared using the catalytic chemical vapor deposition (CCVD) method.

The carbon nanotubes were subjected to purification from impurities in the form of amorphous carbon, soot, and residues of catalysts used in their production process. Purification was carried out using the wet method with a mixture of oxidizing acids, i.e., concentrated nitric acid (V) and concentrated sulfuric acid (VI) in a mass ratio of 3:1. For this purpose, the multiwalled carbon nanotubes were placed in a round-bottom flask and filled with a mixture of acids. The entire mixture was subjected to ultrasonic shaking for 60 min to break up the agglomerates. After shaking the mixture using ultrasound, the nanotubes were quenched in a round-bottom flask under a reflux condenser for a period of 16 h at the boiling point of the oxidizing acid mixture.

Then, the mixture of multiwalled carbon nanotubes in oxidizing acids was filtered on filter paper under reduced pressure and rinsed with distilled water to remove residual acids until the pH value of the filtrate was 6.0. After neutralization of the acid reaction, the carbon nanotubes located on the filter paper were collected and further dispersed in isopropyl alcohol, and the mixture was filtered again. In the final stage, the rinsed carbon nanotubes were dried in a chamber dryer at 50–70 °C for 6 h.

The dried multiwalled carbon nanotubes were mixed with ammonia using a high-efficiency paddle mixer. The entire mixture was then subjected to shaking for 60 min to break up the agglomerate using ultrasound. After ultrasonic shaking, the mixture was left for approximately 24 h. Later, the mixture was filtered again through filter paper, and the collected carbon nanotubes were rinsed several times with demineralized water followed by isopropyl alcohol or ethanol. The resulting precipitate from the filter was collected and placed in an oven for approximately 6–8 h at 60 °C. The dried precipitate was subjected to crushing and grinding.

### 2.2. Preparation of Nanocomposites Based on Epoxy Resins with Carbon Nanotubes

The polymer nanocomposite was obtained using a low viscosity epoxy resin prepared from a mixture of 2,2-bis[4-(2,3-epoxypropoxy)phenyl]propane and 2,3-epoxypropyl o-tolyl ether (Epidian 601, CIECH Sarzyna S.A., Nowa Sarzyna, Poland). The second formulation was based on a mixture obtained from bisphenol AF, epichlorohydrin, and alkyl glycidyl ether (Epidian 652, CIECH Sarzyna S.A., Nowa Sarzyna, Poland). Both resins, hereafter designated 601 and 652, respectively, were crosslinked with isophorone diamine with salicylic acid in benzyl alcohol at a ratio of 1:2 relative to resin 601 (or 652). To obtain better dispersion, compounds improving wettability based on polyamide salts and (trimethoxysilyl)propylamine dispersed in a mixture of 2-butoxyethanol and methanol were used. The aforementioned substances were added to mixture 601 or 652 at a described amount of 1.0–3.0 wt.% relative to the total weight of the composite to improve the deaeration of the mixture. A mixture of polydimethylsiloxanes, cyclosiloxanes, and silica was used as an antifoaming agent. Composites containing additives to improve processability and the degree of dispersing the nanoadditive were designated 652* and 601*.

Using a high-speed nonaerating mixer, the previously mentioned substances were mixed, and multiwalled carbon nanotubes were added at an amount of 0.5 to 2.5 wt.% relative to the total weight of the composite while continuously mixing. Then, after the addition of the hardener, the mixture was mixed for approximately 1–2 min. Afterwards, the mixture was transferred to a vacuum mixer with a helical blade and mixed for another 5 min at maximum speed while thermostating the tank with room temperature water and a vacuum of 0.1 bar. Once all the components of the composite were thoroughly mixed, the material was poured into a mold where crosslinking was carried out. Once the gel point was reached, the molds were transferred to a chamber dryer where crosslinking of the composite was continued at elevated temperature for 8 h.

### 2.3. Structural Analysis of Carbon Nanotubes

Structural analyses were conducted using a JEOL scanning electron microscope (SEM) (model JSM-7800F, JEOL Ltd., Tokyo, Japan). The JEOL JSM-7800F microscope is a high-resolution scanning electron microscope equipped with four detectors: upper electron detector (UED), lower electron detector (LED), backscatter electron detector (BED), and transmission electron detector (TED).

A small amount of the analyzed carbon nanotubes was dispersed in isopropyl alcohol, then the suspension prepared using this method was spotted onto a so-called grid and left under clean conditions to dry. After complete drying, the grid was placed in a holder in the chamber of the microscope. The analysis was performed using the TED detector.

### 2.4. Conductivity Measurements

Material samples were prepared by casting in a cylinder mold. After release, the faces of the samples were ground using fixed sandpaper and a bench drill spindle as a drive. The samples prepared in this way were covered on both sides (parallel surfaces—cylinder bases) with conductive varnish based on silver additive (Electon 40AC, manufactured by Amepox company). To dry and harden the coating, the samples were left at a temperature of 25 degrees for 24 h. After this time, the quality of the surface conductivity was checked by applying the leads of the multimeter at the maximum distance from each other to one of the painted surfaces. The measured values did not exceed 1 Ohm. The prepared samples were placed in the developed measuring holder. It consisted of flat jaws pressed by a spring to the samples. The contacting surfaces were covered by a conductor in the form of copper sheets connected with electrical leads. As a result, a repeatable jaw pressure of approximately 24.5 N was obtained.

The prepared samples were geometrically measured using a mechanical caliper with accuracy to 2 decimal places, which gave a basis for the cross-sectional area and length calculation. A Keysight 34461A Digital Multimeter was used to measure sample resistance. The instrument’s 1 h warm-up procedure and 4-wire configuration were applied to compensate for interferences and the influence of the series resistance of the measurement circuit.

### 2.5. Measurements of Mechanical Properties

**Figure 2:**
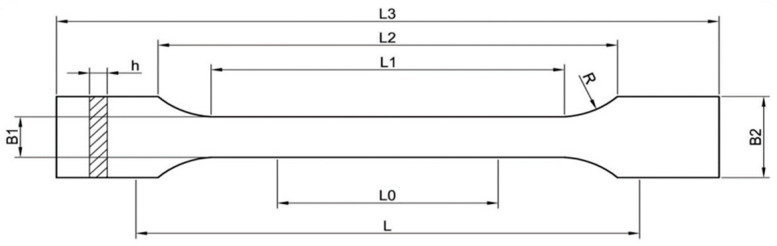
Dimensions of molding.

**Figure 3:**
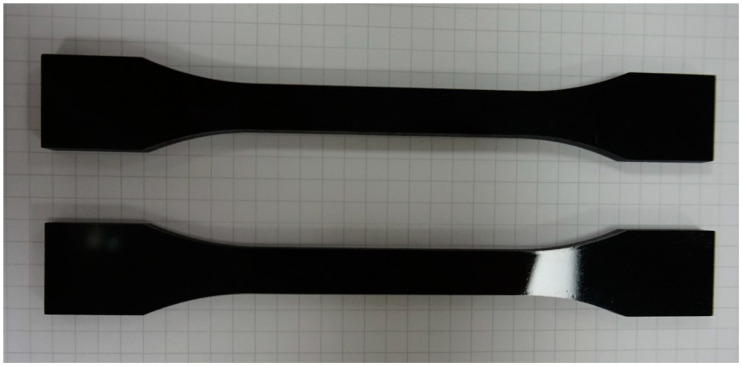
Photographs of moldings for tensile property testing.

Determination of the static tensile properties was conducted according to PN-EN ISO 527:1998 Plastics: Determination of tensile properties [24]. According to the requirements of the standard, the test sample (molding) subjected to the test is flat and has the shape of a paddle as shown in Figure 2. The dimensions of the sample are assumed to be as follows: thickness of 4.0 ± 0.2 mm, measured section width of 10 ± 0.2 mm, and total length over 150 mm. Table 1 shows all the dimensions of the sample used in the study, and Figure 3 shows photographs of the prepared molding.

The tensile property test was carried out using a high precision Shimadzu AG-X Plus 10 kN testing machine equipped with a 10 kN class 1 1/500 load cell with an initial force of 50 N and a crosshead speed of 10 mm/min (Shimadzu Corporation, Tokyo, Japan).

## 3. Results and Discussion

### 3.1. Characterizations of Functionalized CNTs

Figure 3 shows images of functionalized multiwalled carbon nanotubes.

Functionalization of multiwalled carbon nanotubes according to the described procedure allowed the formation of covalent bonds between the functional group and the sidewalls forming the nanotube cylinder [11]. The use of nanotube purification in mixed oxidizing acids resulted in many defect sites on the walls of carbon nanotubes. Defects induced by contact of a strong oxidizing compound with the nanotube are usually stabilized by bonds with carboxyl and/or hydroxyl functional groups, and their presence gives rise to further chemical reactions, including alkylation or arylation, silanization, thiolation, esterification, amidation, and grafting of polymers or biomolecules [1,5].

### 3.2. Measurements of Mechanical Properties

**Figure 4:**
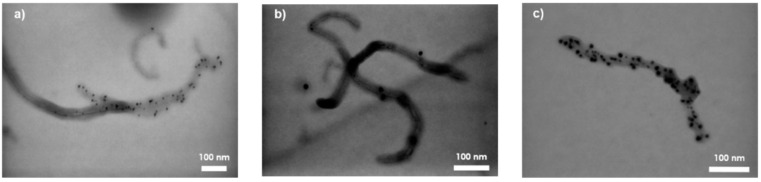
Transmission electron microscopy images of multiwalled carbon nanotubes with deposited amino groups: (**a**) 130,000 times magnification, (**b**) 190,000 times magnification, and (**c**) 200,000 times magnification.

**Figure 5:**
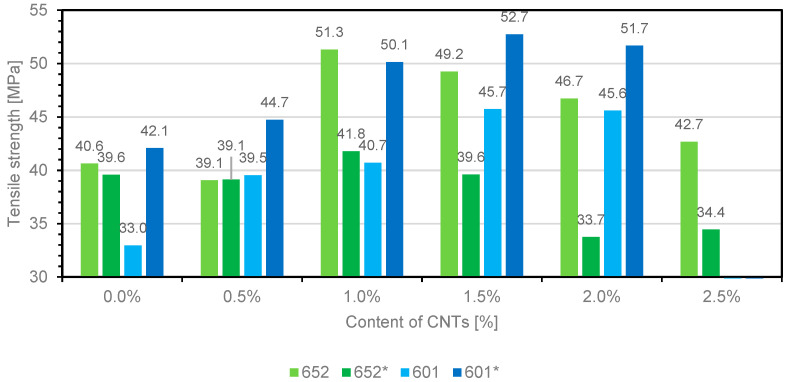
Chart presenting the relationship between the tensile strength of composites with different contents of multiwalled carbon nanotubes (MWCNTs).

**Figure 6:**
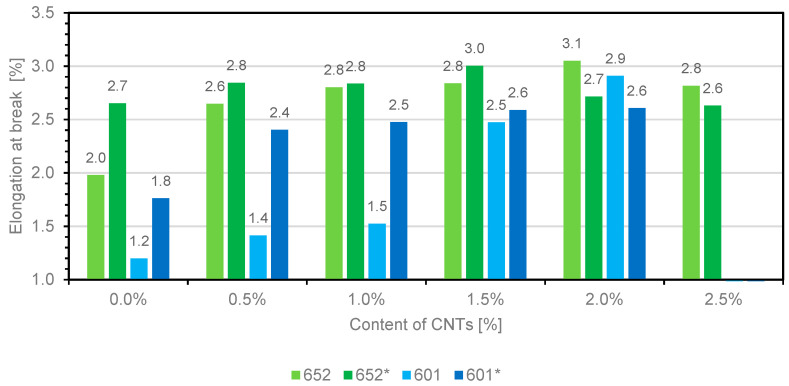
Chart presenting the relationship between the strain and type of composite with various contents of multiwalled carbon nanotubes (MWCNTs).

The study includes the analysis of tensile strength, strain, and Young’s modulus. The lack of results for composites based on 601 resin containing 2.5 wt.% carbon nanotubes is due to the lack of composite samples. The lack of sample is caused by the significant increase in dynamic viscosity of resins in the uncrosslinked state, and the inability to pour the material into a mold. Figure 4, Figure 5 and Figure 6 show the summary of the results obtained.

The analysis of the obtained results indicates that the addition of multiwalled carbon nanotubes in the epoxy composite improves the tensile strength. An increase in tensile strength was observed in composite 652, and the highest values were observed in the sample containing 1 wt.% of carbon nanotubes, which improved the parameter by 26% compared to the reference sample. A higher percentage of filler did not further improve the results obtained; in contrast, a gradual decrease in strength was observed, while 2.5 wt.% of nanotubes did not result in a worse result than the unfilled polymer. An improvement in the properties of polymeric materials can be achieved by filling the material, but after exceeding a certain limit, deterioration of the analyzed parameters is often observed.

Concerning composite 652*, one might note that it has a lower tensile strength than composite 652, and the highest result was obtained when the composite contained 1 wt.% multiwalled nanotubes. The higher proportion of nanofiller resulted in a deterioration of strength, obtaining worse results than the reference sample, which may indicate a high degree of agglomeration of carbon nanotubes.

The use of carbon nanotubes in composite 601 also resulted in the strengthening of the polymeric matrix, and this trend was observed until reaching the maximum strength at 1.5 wt.% filling of the polymeric matrix. Comparable results were obtained for composite 601*, where the addition of substances improving the wettability of the nanoadditive surface ensured better strength parameters.

Figure 5 shows the relationship between the strain and the type of composites with various contents of multiwalled carbon nanotubes (MWCNTs).

The analysis of the above chart shows that, with increasing filler content, the percentage strain of the material increases, reaching an extreme value when the characteristic amount of the additive is used. For composites 652 and 652*, the extremes are reached at 2.0 wt.% and 1.5 wt.% carbon nanotube proportions, respectively, while for composites 601 and 601*, the highest strain values are obtained at the 2 wt.% matrix fill. The use of wetting agents and antifoaming agents based on silicone compounds influenced the increase in strain, which was caused by the higher mobility of polymer chains, which is particularly evident in the results obtained for composites that do not contain nanomaterials. Figure 6 shows the relationship between Young’s modulus and the type of composites with various contents of multiwalled carbon nanotubes (MWCNTs).

The application of multiwalled carbon nanotubes as polymer fillers in epoxy composites alters the Young’s modulus. Deterioration was observed in composites 652 and 652* after the addition of 0.5 wt.% multiwalled nanotubes, although properties similar to, or slightly better than, those of the reference sample were obtained at higher proportions of the nanoadditive. The highest Young’s modulus values were obtained for composite 652 containing 1.5 wt.% carbon nanotubes, while for composite 652*, 1 wt.% carbon nanotubes allowed higher Young’s modulus values to be obtained. Higher contents led to a deterioration of this property. Composites containing modifying additives generally caused the value of Young’s modulus to decrease.

Concerning epoxy 601, when the material was filled with carbon nanotubes up to 1.0 wt.%, no significant change in Young’s modulus was observed. A higher content of nanostructures in the composite caused Young’s modulus values to decrease. A comparable result was observed for epoxy composite 601*, noting that the decrease was observed only when the composite contained 2% carbon nanotubes.

In the case of composite 601, a 20% increase in tensile strength and a 1.3% increase in Young’s modulus value were observed with the addition of 0.5 wt.% carbon nanotubes. To a certain concentration limit, a higher proportion of carbon nanotubes resulted in an increase in tensile strength with a gradual deterioration of Young’s modulus. Comparable correlations were observed for composite 601*.

When measuring composite 652, it was established that, due to the use of 1.5% carbon nanotubes, there was a 26.3% increase in tensile strength and a 1.6% increase in Young’s modulus value. For composite 652*, a 1.0% addition enabled a 4% improvement in mechanical properties, with a 4% increase in Young’s modulus value and a 5.6% increase in tensile strength. Table 2 summarizes the results obtained in the studies described in relation to those reported in previous literature reports.

The obtained results are consistent with the currently available scientific literature data on the use of carbon nanotubes as reinforcement in epoxy composites [14,25,26,27,28]. The results confirm that the addition of multiwalled carbon nanotubes can improve mechanical properties, including tensile strength and Young’s modulus, while increasing the plasticity of the composite under loading [5]. Such dependence is observed up to a certain limit value, and then a gradual or rapid deterioration of the mentioned parameters is observed [29]. In the present work, a significant improvement in tensile strength and a several percent improvement in Young’s modulus were obtained. The obtained results are slightly better than those reported by Gojny et al. [14] with the addition of 0.5 wt.%. MWCNTs (tests were carried out only with carbon nanotube contents of 0.1%, 0.3%, and 0.5%). In most of the literature, the addition of carbon nanotubes has a greater effect on the change in Young’s modulus than the change in tensile strength [11], and parameters obtained through the use of aminofunctionalized multiwalled carbon nanotubes did not give better results than those of previously reported pristine multiwall modified carbon nanotubes used as a reinforcing additive [30].

The increase in tensile strength of polymer composites with 1D nanoadditives results from the transfer of loads occurring in the material to the reinforcing phase. Considering carbon nanotubes, this effect is caused by a large surface area and covalent bond formation between polymer chains. The reinforcement effect can be achieved by arranging polymer chains along the carbon nanotubes in the axial direction. This method of arranging the filler may be achieved by the presence of amine groups on the carbon nanotube surface, which leads to a reduction in the average length of the polymer chain, as well as an improvement in the cross-linking of polymer chains. As a result, an increase in tensile strength and longitudinal tensile modulus can be achieved.

The analysis of the obtained results shows that no significant changes in the Young’s modulus are observed. In the case of the tested composites, the condensation polymerization process was carried out until a specific viscosity of resin was achieved. At the same time, the shortening of the carbon nanotube structure caused by chemical functionalization reduced the interface between the polymer matrix and the dispersed phase, which resulted in no improvement or even deterioration of the elastic modulus.

### 3.3. Results of Direct Current Resistivity Measurements

**Figure 7:**
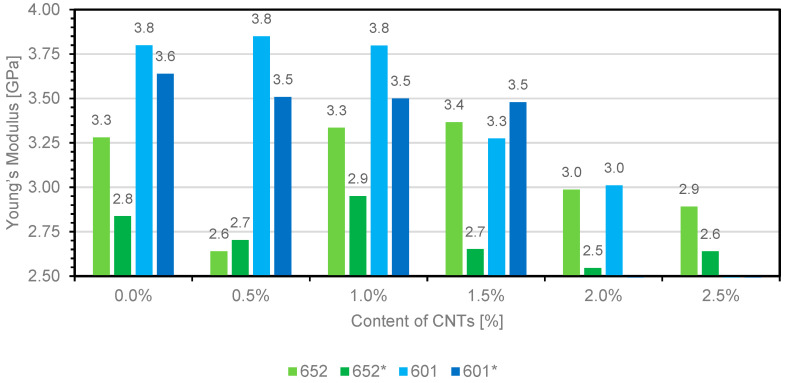
Chart presenting the relationship between Young’s modulus and the type of composites with various contents of multiwalled carbon nanotubes (MWCNTs).

The resistivity is measured using an indirect method by measuring the vertical resistivity, taking into account the surface area and thickness of the sample. In this study, the resistivity of cylindrical samples with dimensions of approximately 10 mm in height and approximately 12 mm in base diameter was measured. The measurements of the resistivity of the samples were recalculated based on their exact dimensions, thus obtaining the resistivity values, which are summarized in the chart below depending on the mass fraction of carbon nanotubes in the finished composite. Two measurements were performed for each sample, and the mean value is presented (Figure 7).

**Figure 8:**
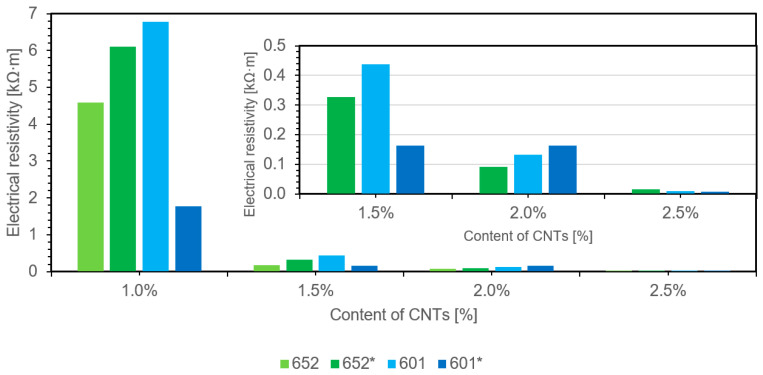
Chart presenting the relationship between the resistivity and weight fraction of multiwalled carbon nanotubes for the composites studied.

The study indicates that the addition of multiwalled carbon nanotubes reduces the resistivity of the material, thus improving its conductivity (Figure 8). For composites not containing carbon nanotubes, no results were obtained as, due to their dielectric properties, they were beyond the measurement range of the device. For all the epoxy composites tested, resistivity results were recorded at a 1 wt.% content of multiwalled carbon nanotubes, with 1.5 percent nanoadditive causing a significant increase in electrical conductivity. The addition of large amounts of carbon nanotubes to the polymer matrix results in further improvement in conductivity, reaching a plateau.

Comparative studies indicate that additives improving the processing and dispersion of carbon nanotubes worsened the electrical conductivity in composites based on sample 652. The opposite effect was obtained for composite 601, therefore one might assume that the selected components for this polymer matrix improved the degree of dispersion of the carbon additive. The greatest effect was obtained by the additive in composite 601. The measured resistivity is generally lower in composites based on 601, which may be related to the higher ability to form crosslinks that allow charge flow. Table 3 summarizes the results obtained in the studies, described in relation to those reported in previous literature reports.

The obtained results are consistent with the currently available literature data on the use of carbon nanotubes in epoxy composites [20,21,28,31,32,33,34,35,36,37], while for nanotubes functionalized with amines, slightly worse results than those reported were obtained [23]. The surface functionalization of carbon nanotubes is crucial concerning the electrical conductivity of polymer nanocomposites by facilitating the dispersion of the additive and the formation of uniformly distributed conductive crosslinks, which ultimately lowers the percolation threshold. However, it is worth noting that excessive modification introducing many heterogeneous atoms on the surface causes electron flow disturbances, thus degrading the electrical properties resulting from the addition of carbon nanotubes alone [1]. The lower values of measured electrical conductivities are probably caused by chemical treatment with strong oxidizing acids and the use of ultrasound, which shortens the length of carbon nanostructures through their excessive degradation. Purification and functionalization based on the use of strongly oxidizing acids, e.g., sulfuric acid or nitric acid, can cause degradation of the structure and consequently, their shortening and change in the length-to-diameter ratio. Both of these changes adversely affect the electrical conductivity obtained [42].

The conclusions drawn are confirmed by the illustrative photos in Figure 4, where the lengths of the nanotubes are smaller than the dimensions of the structures declared by the manufacturer before the functionalization process. The reduction of the aspect ratio made it necessary to use a higher mass fraction of carbon filler to obtain a conductive network in the polymer matrix with dielectric properties. To obtain the same order of electrical conductivity values as those obtained with 1 wt.% addition of carbon nanotubes in the works of [21,28,32], it was necessary to use 2.5 wt.% carbon nanotubes functionalized with amine groups.

## 4. Conclusions

This paper presents the effect of multiwalled carbon nanotubes containing amine groups on their surfaces on the electrical and mechanical properties of epoxy composites. The addition of carbon nanotubes allowed us to obtain an electrically conductive material, in which nanotubes form a network for the charge flowing through the material. For all materials, the percolation threshold was obtained with 1% addition of multiwalled carbon nanotubes, and filling the polymer with a higher content of carbon nanotubes increased its conductivity. Carbon nanotube purification in a mixture of oxidizing acids strongly defected the nanotube structure. This allowed the formation of multiple bonds at the defect sites, allowing the grafting of functional groups, thereby increasing the compatibility with the polymer matrix and obtaining a better degree of dispersion. However, the defect may have contributed to shortening the length of the carbon nanotubes, which resulted in poorer percolation thresholds with respect to the literature data.

The use of carbon nanotubes as polymer reinforcement allows higher values of tensile strength and a higher strain percentage to be achieved. In contrast, Young’s modulus values did not increase significantly, and higher nanofiller percentages resulted in a drastic decrease in the values of the abovementioned properties.

An increase in tensile strength was achieved when the weight fraction of nanotubes did not exceed 1.5 wt.% for composite 652* and 2.0 wt.% for composites 652, 601, and 601*. The addition of larger amounts of carbon filler caused the deterioration of properties, and the achieved effect is characteristic for polymer composites where nanomaterials of linear and layered structures were added. This is due to the formation of aggregates and agglomerates of carbon nanotubes, which may result in a significant increase in viscosity of the uncross-linked resin, and thus hinder dispersion of the additive in the polymer (macroscopic dispersion) and disentanglement of carbon nanotubes (nanoscopic dispersion). The compounds proposed can be used to obtain a material with uniformly dispersed carbon nanotubes.

Due to the difficulty in achieving a high degree of homogenization of carbon nanotubes in an epoxy matrix, the strength parameters of the composites are often lower than expected.

## Figures and Tables

**Table 1:** Dimensions of molding for tensile property testing according to the EN-ISO 527-1 standard.

Dimensions of Molding	Type B1
L3—total length	150 mm
L1—length of the designated section	40 mm
R—radius	60 mm
L2—distance between parallel sections	106 mm
B2—width at ends	20 mm
B1—width at narrow section	10 mm
H—recommended thickness	4 mm
L0—measured length	50 mm
L—initial distance between handles	115 mm

**Table 2:** Mechanical properties of pure epoxy and CNT/epoxy composites.

CNT Type	CNT WeightFraction	Young’s Modulus		Tensile Strength		Ref.
	[%]	[GPa]	ΔE [%]	[MPa]	Δσ [%]	
652 with no filler	0.0	3.28	-	40.64	-	Present work
MWCNT-NH_2_	0.5	2.64	−19.5	39.06	−3.9	
	1.0	3.33	1.6	51.31	26.3	
	1.5	3.37	2.6	49.24	21.2	
	2.0	2.99	−8.9	46.73	15.0	
	2.5	2.89	−11.9	42.67	5.0	
652* with no filler	0.0	2.84	-	39.58	-	
MWCNT-NH_2_	0.5	2.70	−4.7	39.12	−1.2	
	1.0	2.95	4.0	41.79	5.6	
	1.5	2.65	−6.5	39.61	0.1	
	2.0	2.55	−10.3	33.74	−14.8	
	2.5	2.64	−7.0	34.44	−13.0	
601 with no filler	0.0	3.80	-	32.96	-	
MWCNT-NH_2_	0.5	3.85	1.3	39.54	20.0	
	1.0	3.80	−0.1	40.71	23.5	
	1.5	3.27	−13.8	45.73	38.8	
	2.0	3.40	−20.8	45.59	38.8	
	2.5	-	-	-	38.3	
601* with no filler	0.0	3.64		42.07	-	
MWCNT-NH_2_	0.5	3.51	−3.6	44.74	6.3	
	1.0	3.50	−3.8	50.13	19.1	
	1.5	3.48	−4.4	52.73	25.3	
	2.0	-	-	51.67	22.8	
	2.5	-	-	-	-	
Epoxy with no filler	0.0	1.48	-	46.46		[25]
MWCNT	0.5	1.68	13.5	50.25	8.2	
	1.0	1.87	26.4	58.65	26.2	
	3.0	1.69	14.2	54.48	17.3	
Epoxy with no filler	0.0	2.60	-	63.80		[14]
MWCNT	0.1	2.78	6.9	62.97	−1.3	
	0.3	2.77	6.5	63.17	−1.0	
	0.5	2.61	0.4	61.52	−3.6	
Epoxy with no filler	0.0	2.60		63.80		[14]
MWCNT-NH_2_	0.1	2.88	10.8	64.67	1.4	
	0.3	2.81	8.1	63.64	−0.3	
	0.5	2.82	8.5	64.27	0.7	
Epoxy with no filler	0.0	1.21	-	26.00	-	[26]
Untreated CNTs	1.0	1.38	14.0	42.00	61.5	
Acid treated CNTs	1.0	1.22	0.8	44.00	69.2	
Amine treated CNTs	1.0	1.23	1.7	47.00	80.8	
Plasma treated CNTs	1.0	1.61	33.1	58.00	123.1	
Epoxy with no filler	0.0	1.18	-	52.00	-	[27]
MWCNT	2.0	1.18	0.0	46.00	−11.5	
MWCNT-PVA	2.0	1.35	14.4	55.00	5.8	
MWCNT-AEO9	2.0	1.39	17.8	62.00	19.2	
MWCNT-AEO7	2.0	1.34	13.6	58.00	11.5	

**Table 3:** Electrical conductivity of the nanocomposites as a function of filler content in weight percent.

CNT Type	Content of CNTs	Processing Method	Electrical Resistivity	Electrical Conductivity	Ref.
	[%]		[Ω·m]	[S/m]	
652 with no filler	0.0	Vacuum stirred	-	-	Present work
MWCNT-NH_2_	0.5		-	-	
	1.0		4.60 × 10^3^	2.18 × 10^−4^	
	1.5		1.70 × 10^2^	5.88 × 10^−3^	
	2.0		7.35 × 10^1^	1.36 × 10^−2^	
	2.5		9.43 × 10^0^	1.06 × 10^−1^	
652* with no filler	0.0	Vacuum stirred	-	-	
MWCNT-NH_2_	0.5		-	-	
	1.0		6.11 × 10^3^	1.64 × 10^−4^	
	1.5		3.28 × 10^2^	3.05 × 10^−3^	
	2.0		9.23 × 10^1^	1.08 × 10^−2^	
	2.5		1.62 × 10^1^	6.18 × 10^−2^	
601 with no filler	0.0	Vacuum stirred	-	-	
MWCNT-NH_2_	0.5		-	-	
	1.0		6.78 × 10^3^	1.47 × 10^−4^	
	1.5		4.37 × 10^2^	2.29 × 10^−3^	
	2.0		1.33 × 10^2^	7.52 × 10^−3^	
	2.5		-	-	
601* with no filler	0.0	Vacuum stirred	-	-	
MWCNT-NH_2_	0.5		-	-	
	1.0		1.77 × 10^3^	5.66 × 10^−4^	
	1.5		1.63 × 10^2^	6.13 × 10^−3^	
	2.0		1.63 × 10^2^	6.13 × 10^−3^	
	2.5		8.62 × 10^0^	1.16 × 10^−1^	
MWCNT	1.0	Stirred, heat sheared (slowly)	2.50 × 10^0^	4.00 × 10^−1^	[21]
MWCNT	1.0	Stirred, heat sheared (medium)	3.33 × 10^0^	3.00 × 10^−1^	
MWCNT	0.6	Stirred, heat sheared (fast)	2.50 × 10^1^	4.00 × 10^−2^	
MWCNT	0.3	Calendered, stirred	1.00 × 10^2^	1.00 × 10^−2^	[31]
MWCNT NH_2_-functionalized	0.4	Calendered, stirred	2.00 × 10^3^	5.00 × 10^−4^	
MWCNTs	0.5	Calendered, stirred	1.00 × 10^2^	1.00 × 10^−2^	
MWCNT	1.0	Sonicated	5.00 × 10^0^	2.00 × 10^−1^	[28]
MWCNT	1.0	Stirred	5.00 × 10^1^	2.00 × 10^−2^	
MWCNTs	10.0	Solution mixing	3.33 × 10^2^	3.00 × 10^−3^	[32]
SDS suspended MWCNTs	0.5	Bulk mixing	4.00 × 10^6^	2.50 × 10^−7^	[33]
MWCNTs	2.5	Solution mixing	7.69 × 10^1^	1.30 × 10^−2^	[34]
MWCNTs	1.4	Solution mixing	2.00 × 10^0^	5.00 × 10^−1^	[35]
Oxidized MWCNTs	1.0	Solution mixing	1.00 × 10^2^	1.00 × 10^−2^	[36]
Pristine MWCNTs	1.0	Solution mixing	1.00 × 10^2^	1.00 × 10^−2^	[37]
MWCNTs	5.0	Calendered, stirred	2.00 × 10^−2^	5.00 × 10^1^	[38]
MWCNT	1.0	Heat sheared	5.00 × 10^−1^	2.00 × 10^0^	[20]
MWCNT	0.5	Heat sheared	2.50 × 10^0^	4.00 × 10^−1^	[39]
MWCNT	3.0	Stirred	2.00 × 10^−1^	5.00 × 10^0^	[40]
MWCNT	10.0	Stirred	2.00 × 10^2^	5.00 × 10^−3^	[32]
MWCNT	4.0	Stirred, hot pressed	2.00 × 10^−1^	5.00 × 10^0^	[41]
MWCNT	2.0	Calendered, vacuum stirred	3.33 × 10^1^	3.00 × 10^−2^	[42]

## Data Availability

The data underlying this article will be shared on reasonable request from the corresponding author.
